# Adherence of Mexican physicians to clinical guidelines in the management of breast cancer: Effect of the National Catastrophic Health Expenditure Fund

**DOI:** 10.1371/journal.pone.0212841

**Published:** 2019-03-20

**Authors:** Carmelita E. Ventura-Alfaro, Leticia Ávila-Burgos, Gabriela Torres-Mejía

**Affiliations:** 1 Departament of Epidemiology, Mexican Institute of Social Security, Guadalajara, Jalisco, Mexico; 2 Center for Health Systems Research, National Institute of Public Health, Cuernavaca, Morelos, Mexico; 3 Center for Population Health Research, National Institute of Public Health, Cuernavaca, Morelos, Mexico; Flinders University, AUSTRALIA

## Abstract

**Aim:**

To assess the adherence of physicians to the Medical-Care Guidelines for Malignant Breast Tumors in Mexico, before and after the allocation of federal subsidies from the Catastrophic Health Expenditure Fund (*FPGC* by its Spanish initials) to accredited hospitals, a strategy implemented with the view of offering free treatment to women with breast cancer (BC).

**Material and methods:**

Based on a cross-sectional design, we gathered information on 479 BC patients who had been attended to at in four *FPGC*-accredited hospitals. Analysis centered on those treated within either three years before or three years after the accreditation of their attending hospitals. The four hospitals analyzed were located in the North, South, West and Center of the country. Information on all medical procedures performed during treatment was drawn from hospital medical records. Information on the socio-demographic characteristics of the patients was obtained by means of face-to-face interviews conducted in their homes.

**Results:**

Adherence of physicians to the Guidelines grew by 12.8 percent (from 43.4 to 56.2 percent) after *FPGC* accreditation (*p*<0.001) and varied according to the clinical stage of the disease, with much lower levels of adherence observed in the advanced stages (*p*<0.05).

**Conclusions:**

The *FPGC* strategy increased the adherence of physicians to the Medical-Care Guidelines for Malignant Breast Tumors in Mexico.

## Introduction

The most prevalent type of oncological disease in women, breast cancer (BC), has become a worldwide public health concern. Of the 20,000 new cases reported in Mexico each year, one quarter are fatal [1]. BC treatment has improved considerably over time; however, inadequate adherence of medical personnel to the Medical-Care Guidelines for Malignant Breast Tumors established by the Mexican Social Protection System in Health (*SPSS* by its Spanish initials) poses a challenge to the Mexican Health-Care System, characterized by limited resources and burdened by the inordinately high costs of BC diagnosis and treatment [2, 3].

By law, since 2007, all public hospitals in Mexico treating BC patients receive support from the federal Catastrophic Health Expenditure Fund (*FPGC* by its Spanish initials) as a strategy for offering free health services to women with BC. Prior to 2007, public health services for the treatment for BC were limited to women with health insurance from institutions such as the Mexican Institute of Social Security, which benefits only individuals employed in the formal sector [4]. In 2004, the *SPSS*, a voluntary public health insurance program, was created in order to provide access to health-care services and financial protection to all those without Social Security coverage. The *SPSS* manages two major operations: (a) the *Seguro Popular*, its key component, offers a package of primary- and secondary-care services to its beneficiaries who [5], by 2017, had reached 60.5 million, or 43.2% of the Mexican population [6]; and (b) the *FPGC* finances expensive health-care treatments with a view to reducing the economic strain imposed by disease on the poorest sectors of the population [5, 7]. Incorporated as part of the *FPGC* catalog since 2007 [2], BC treatment is provided to women free of charge through the intermediary of hospitals accredited on the basis of criteria concerning capacity, safety and quality in patient care [8]. With the aim of improving quality, optimizing resources and minimizing heterogeneity in the treatment of BC, all relevant practices have been standardized and medical guidelines developed for its diagnosis and treatment [9]. Adherence to medical guidelines is a complex task, however, particularly for BC, a chronic disease requiring surgery and adjuvant treatments such as radiotherapy, chemotherapy, immunotherapy and/or hormonotherapy, depending on the specific characteristics of the tumors and patients [10].

The level of adherence to medical-care guidelines designed for oncology patients varies from 20 to 100 percent in most countries [11–20]. Long-term consequences of non-adherence commonly include poor quality of care, reduced effectiveness, increased mortality, a decline in the health status of the population, higher health costs and underutilization of limited health resources [13, 21, 22]. Evaluating adherence to medical-care guidelines is necessary for planning effective and efficient therapy, but most importantly, for establishing whether health outcomes are attributable to the treatment implemented [13, 22].

The objective of this study was to assess the level of adherence of physicians to the *SPSS* Medical-Care Guidelines for Malignant Breast Tumors before and after hospitals were accredited to receive *FPCG* financing as a mechanism for providing BC treatment free of charge. We hypothesized that the level of adherence to the *SPSS* Guidelines had improved after the *FPGC* accreditation of hospitals.

## Material and methods

### Study design

A cross-sectional design was used to obtain information on BC patients who had undergone treatment within either three years before or three years after their attending hospitals were accredited to receive *FPGC* financing.

### Setting and study population

**Fig 1:**
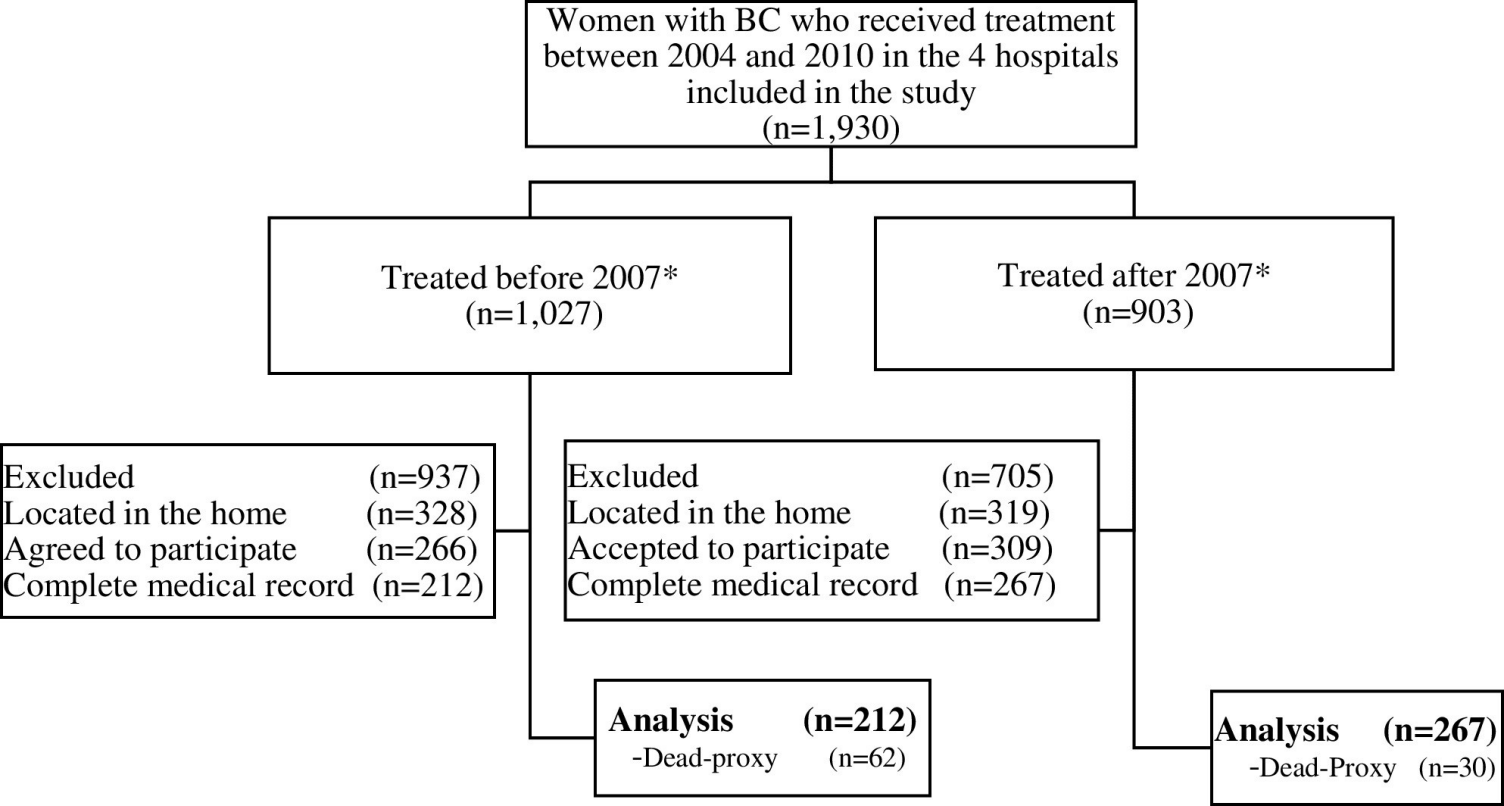
Participant flow. * 2007, year in which the sampled hospitals were accredited to receive *FPGC* financing.

We recruited BC patients from four hospitals in the North, South, West and Center of the country. All 32 states in Mexico have at least one *FPGC-*accredited hospital for BC care. The criteria for selecting the hospitals in our sample revolved around those with the highest possible concentration of BC cases, including facilities in poor states. Three of the hospitals in our sample were located in states accounting for 48.8 percent of BC cases in Mexico; the fourth was located in a state characterized by high poverty levels. All four had been receiving *FPGC* support since 2007. Overall, these hospitals had treated 1,027 BC patients prior to and 903 following *FPGC* accreditation; however, given the financial limitations of our study, we confined our analysis to patients residing within a distance equivalent to one hour’s drive from the hospitals: of these, 328 had been treated before and 319 after accreditation, 575 (88.9 percent) agreed to participate in the study, and a final sample of 479 (74 percent) were able to provide complete medical records (Fig 1).

### Data sources

We conducted face-to-face interviews with women who had been treated for BC in the selected hospitals. The interviews were based on a questionnaire concerning socio-demographic characteristics and were held in their homes. All medical information regarding the management of their disease (clinical stage, type of surgery and adjuvant treatments such as radiotherapy, chemotherapy, immunotherapy and/or hormonotherapy) was drawn from their medical records.

### Outcome variable

*Level of adherence of physicians to the SPSS Medical-Care Guidelines for Malignant Breast Tumors*. Our outcome variable was expressed as a numeric value representing the relationship between the number of procedures undergone by each BC patient in a timely and accurate manner and the total number of procedures stipulated in the *SPSS* Guidelines for the clinical stage of her condition. This value was estimated within a continuous interval [0,1], where 0 represented 0 percent adherence to the *SPSS* Guidelines and 1, 100 percent adherence. We used the following equation to construct the level of adherence for each patient:

$$LA_{i}=\frac{\sum_{k=1}^{m}X_{ik}}{\sum_{j=1}^{n}X_{ij}}$$ (1)

Where, LA = Level of adherence, *i* = Patient at clinical stage *i*, *j* = Procedures required for clinical stage *i*, and *k* = Procedures followed accurately and in a timely manner during clinical stage *i*.

**Fig 2:**
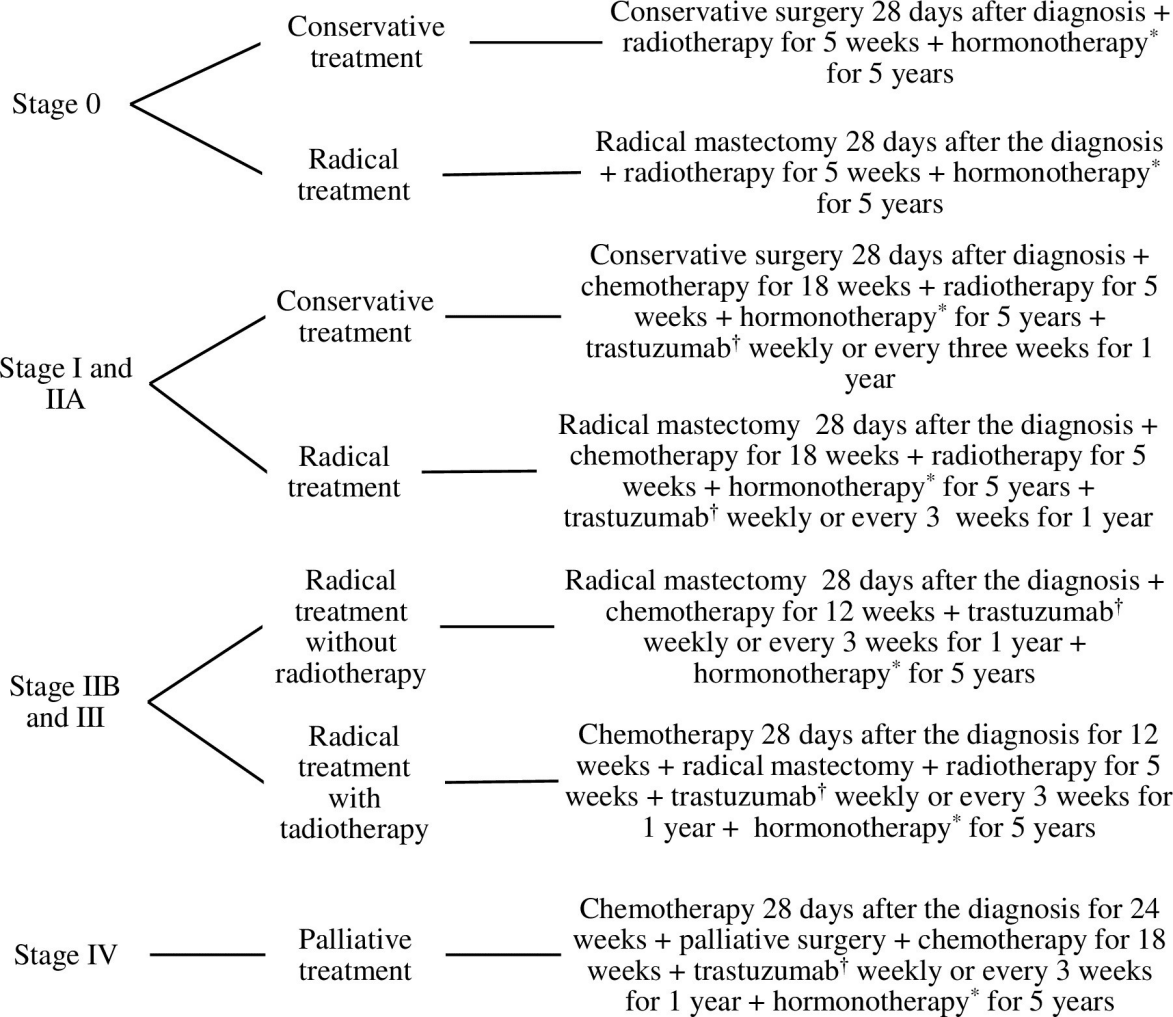
Treatment of breast cancer by clinical stage, according to the *SPSS* Medical-Care Guidelines for Malignant Breast Tumors. *With estrogen receptors (+). † With HER2neu receptors (+).

*Adherence to the Guidelines* denoted the degree of compliance of attending physicians with the *SPSS* Medical-Care Guidelines for Malignant Breast Tumors [9], according to the clinical stage of the disease in each patient. We constructed an adherence index based on the six medical procedures specified in the *SPSS* Guidelines: surgery; radiotherapy (2Gy daily for 5 weeks); chemotherapy (four cycles of fluoracyl, adriamycin and cyclophosphamide, followed with paclitaxel weekly for 12 weeks); adjuvant chemotherapy (steroids and antiemetic treatment); hormonotherapy; and/or trastuzumab medication in eligible patients[3, 9]. These procedures were assessed according to the clinical stage of the disease and to the time of use, as established by the *SPSS* Medical-Care Guidelines for Malignant Breast Tumors [9] (see Fig 2). Each procedure was categorized dichotomously as follows: 0 = *no adherence* was determined when the procedure was not followed, was not included in the *SPSS* Guidelines, or was performed as specified in the Guidelines but at the wrong time; 1 = *adherence* was determined when the procedure was included in the *SPSS* Guidelines and was performed within the indicated time frame.

### Independent variable

Our independent variable was *FPGC* financing provided to accredited hospitals since 2007 as a strategy for offering treatment to BC patients free of charge. Our analytic sample included two groups of women who had been treated before and after 2007.

### Covariates

We classified the clinical stages of BC in our sample of patients as follows: *Stage 0*: Tis, N0, M0; *Stage I and IIA*: T1, N0, M0 / T0, N1, M0 / T1, N1, M0 / T2 N0, M0; *Stage IIB and III*: T2, N1, M0 / T3, N0, M0 / T0, N2, M0 / T1, N2, M0 / T2, N2, M0 / T3, N1, M0 / T3, N2, M0 / T4, any N, M0 / any T, N3, M0; and *Stage IV*: any T, any N, M1 [23].The following socio-demographic variables were included in our analytical models: age (<40, 40–49, 50–59, 60–69 and ≥70 years); schooling (none, elementary, junior high school, high school and professional); marital status (consensual union, separated/widow/divorced, married, and single), and paid work (yes/no).

### Statistical analysis

We performed an exploratory analysis of the variables of interest and two bivariate analyses of BC patients before and after *FPGC* implementation: the first bivariate analysis used proportions and χ^2^ tests to identify statistically significant differences among the characteristics of the two groups; the second considered the adherence index, estimating means and 95 percent confidence intervals (CI).

In order to facilitate adjustment, and given that our outcome variable (level of adherence) had a value ranging from 0 to 1, we used the parametric beta regression model for variance-robust cluster estimation (four states), as proposed by Salinas-Rodriguez et al. [24]. As this model excludes 0s and 1s, we re-escalated these values, increasing or reducing the value 0.001, respectively. We employed graphic criteria for goodness of fit tests [25] and Cook’s distance and leverage points [26] to identify the presence of influential observations; none of relevance to our analysis emerged. Estimates were performed using Stata SE v10.0 [27] and R [28] statistical packages.

### Ethical issues

Our study was approved by the Ethics, Biosafety and Research Committees of the National Institute of Public Health, NIPH (Reference Number 774). We obtained prior oral consent from participants for collecting their socio-demographic data by means of a face-to-face questionnaire, for reviewing their medical records and for calling them if further information were required. During the consent procedure, we informed potential participants that the information collected would be handled in a strictly confidential manner and that they were free to refuse to participate or to withdraw from participating in the study at any time. We also assured them that the decision to participate or to refrain from participating would in no way affect their services at the hospital. A card was handed to the patients listing the names and telephone numbers of the President of the NIPH Ethics Committee and the Principal Investigator in the study. The NIPH Ethics Committee determined that it was unnecessary for us to obtain written informed consent given that our study involved minimal risk and did not entail biological sampling or include minors (the minimum age of participants was 29 years).

## Results

We recruited a total of 479 women between the ages of 29 and 99 years: 212 had been treated for BC before and 267 after their hospitals were accredited to receive *FPGC* financing. Their mean ages were 54.3 years (SD 12.1 years) and 56.2 years (SD 13.6 years), respectively. As shown in Table 1, the two groups exhibited similar socio-demographic characteristics: 28% of participants had no schooling and slightly over half were married. The clinical stages of their condition were distributed similarly as well, with the majority of patients (60%) in stages IIB and III. In comparing the levels of adherence of physicians to the *SPSS* Guidelines before and after 2007, the former came out lower (p<0.001). The proportion of women who received chemotherapy (p = 0.012), trastuzumab medication (p<0.001) and estrogen (p<0.001) or Her2/neu (p<0.001) receptor tests was greater among those treated after *FPGC* accreditation.

**Table 1:** Socio-demographic characteristics and clinical stages of women with breast cancer: Comparison of the groups treated before and after *FPGC*^a^ accreditation of the four participating hospitals, Mexico^b^.

	Before *FPGC*^a^		After *FPGC*^a^		Total		P Value
	n = 212		n = 267		n = 479		
	n	%	n	%	n	%	
**Characteristics of women**							
**Clinical stage of breast cancer**							
0	2	0.9	6	2.2	8	1.7	0.134
I and IIA	59	27.8	97	36.3	156	32.6	
IIB and III	139	65.6	150	56.2	289	60.3	
IV	12	5.7	14	5.2	26	5.4	
**Age groups**							
< 40	16	7.5	22	8.2	38	7.9	0.266
40–49	60	28.3	82	30.7	142	29.6	
50–59	66	31.1	82	30.7	148	30.9	
60–69	31	14.6	50	18.7	81	16.9	
≥70	39	18.4	31	11.6	70	14.6	
**Schooling**							
None	64	30.2	69	25.8	133	27.8	0.121
Elementary	64	30.2	67	25.1	131	27.3	
Junior high school	32	15.1	64	24.0	96	20.0	
High school	31	14.6	45	16.8	76	15.9	
Professional	21	9.9	22	8.2	43	9.0	
**Marital status**							
Consensual union	17	8.0	16	6.0	33	6.9	0.196
Separated/divorced/widow	43	20.3	58	21.7	101	21.1	
Married	117	55.2	130	48.7	247	51.6	
Single	35	16.5	63	23.6	98	20.5	
**Paid work**							
No	122	57.5	153	57.3	275	57.4	0.957
Yes	90	42.4	114	42.7	204	42.6	
**Treatment characteristics**							
**Conservative surgery**							
No	174	82.1	232	86.9	406	84.8	0.145
Yes	38	17.9	35	13.1	73	15.2	
**Radical surgery**							
No	78	36.8	78	29.2	156	32.6	0.079
Yes	134	63.2	189	70.8	323	67.4	
**Chemotherapy**							
No	50	23.6	39	14.6	89	18.6	0.012
Yes	162	76.4	228	85.4	390	81.4	
**Radiotherapy**							
No	82	38.7	100	37.4	182	38.0	0.784
Yes	130	61.3	167	62.5	297	62.0	
**Estrogen receptors**							
No	48	22.6	16	6.0	64	13.4	<0.001
Yes	164	77.4	251	94.0	415	86.6	
**Hormonotherapy**							
No	101	47.6	116	43.4	217	45.3	0.360
Yes	111	52.4	151	56.5	262	54.7	
**Her2/neu receptors**							
No	58	27.4	21	7.9	79	16.5	<0.001
Yes	154	72.6	246	92.1	400	83.5	
**Trastuzumab**							
No	207	97.6	224	83.9	431	89.9	<0.001
Yes	5	2.4	43	16.1	48	10.1	
**Characteristics of medical care**							
**Hospital**							
South	6	2.8	12	4.5	18	3.8	0.001
North	78	36.8	115	43.1	193	40.3	
West	26	12.3	56	21.0	82	17.1	
Center	102	48.1	84	31.5	186	38.8	

^a^*FPGC*: Spanish acronym for Catastrophic Health Expenditure Fund

^b^Bivariate analysis included proportions and χ^2^ tests in order to evaluate the statistical significance between the exposed and unexposed groups

Table 2 shows the relationships between estrogen and Her2/neu receptor tests performed and the therapy specified under the *SPSS* Guidelines. As can be noted, 6.2 percent of women in the study received trastuzumab medication before and 40.6 percent after *FPGC* accreditation (*p*<0.001). Furthermore, several women who obtained negative Her2/neu receptor results or did not undergo receptor testing received trastuzumab treatment although it was not required in the Guidelines.

**Table 2:** Relationship between receptor (estrogen and HER2/neu) testing and required therapy in women with breast cancer: Comparison of the groups treated before and after *FPGC*^a^ accreditation of the four participating hospitals, Mexico.

	Before *FPGC*^a^			After *FPGC*^a^			Total		
	n = 212			n = 267			n = 479		
**Test/Therapy**	**Estrogen Receptors**								
**Hormonotherapy**	**Positive**	**Negative or NP**^**b**^	**p value**	**Positive**	**Negative or NP**^**b**^	**p value**	**Positive**	**Negative or NP**^**b**^	**p value**
	(n = 95)	(n = 117)		(n = 162)	(n = 105)		(n = 257)	(n = 222)	
Received	86.3%	24.8%	<0.001	82.1%	17.1%	<0.001	83.7%	21.2%	<0.001
	**Her2/neu Receptors**								
**Trastuzumab**	**Positive**	**Negative or NP**^**b**^	**p value**	**Positive**	**Negative or NP**^**b**^	**p value**	**Positive**	**Negative or NP**^**b**^	**p value**
	(n = 65)	(n = 147)		(n = 101)	(n = 166)		(n = 166)	(n = 313)	
Received	6.2%	0.7%	0.015	40.6%	1.2%	<0.001	27.1%	1.0%	<0.001

^a^*FPGC*: Spanish acronym for Catastrophic Health Expenditure Fund

^b^NP: not performed

The number of participants for which medical records showed over 70 percent adherence to the *SPSS* Guidelines was 18.4 percent and 20.2 percent before and after *FPGC* accreditation, respectively (S1 Table). The mean level of adherence in the overall study population was 46.2 percent. Before and after *FPGC* accreditation, the mean levels of adherence were 42 percent and 49.6 percent respectively (*p*<0.001). For all participants, the level of adherence was higher in the earlier stages of the disease, including cancer *in situ* (62.5 percent), than in later stages such as stage IV (30.8 percent) (S1 Table).

Beta regression modeling revealed that, prior to *FPGC* accreditation, the estimated mean level of adherence of physicians, according to the index based on information from the medical records, was 33.2 percent (constant value in Table 3). This increased significantly to 69.3 percent for those treated after *FPGC* accreditation (*p*<0.001) (Table 3). These proportions differ from the means shown in S1 Table because they were adjusted for potential confounders. The estimated mean level of adherence of physicians increased 6.9 times (*p* = 0.013) in women at clinical stage 0, compared with those reported at clinical stage IV; three times for clinical stages I and IIA (*p*<0.001), and 63.4 percent (*p* = 0.131) for clinical stages IIB and III (Table 3).

**Table 3:** Factors associated with the level of adherence of physicians^a^ to the *SPSS* Medical-Care Guidelines for Malignant Breast Tumors: Comparison of the groups of women treated before and after *FPGC*^b^ accreditation of the four participating hospitals, Mexico.

	RPR^c^	[CI 95% ]	p value	CEM^d^	[CI 95% ]	p value
***FPGC***^**b**^	1.693	[1.346–2.130]	<0.001	0.128	[0.073–0.183]	<0.001
**Clinical stage of breast cancer**						
0	6.898	[1.512–31.468]	0.0013	0.410	[0.199–0.622]	<0.001
I and IIA	3.170	[2.229–4.508]	<0.001	0.280	[0.199–0.362]	<0.001
IIB and III	1.634	[0.864–3.090]	0.131	0.119	[-0.032–0.271]	0.123
IV	1.0					
**Hospital**						
South	0.298	[0.269–0.331]	<0.001	-0.252	[-0.267 –-0.238]	<0.001
North	0.694	[0.630–0.764]	<0.001	-0.089	[-0.113 –-0.066]	<0.001
West	1.231	[1.021–1.483]	0.029	0.051	[0.005–0.096]	0.030
Center	1.0					
**Constant**	0.332	[0.200–0.550]	<0.001	**Predicted proportion**: 0.434		

Number of observations: 479

^a^A beta regression model was used because the dependent variable (level of adherence of physicians) had a value ranging from 0 to 1. Level of adherence was expressed as a numeric value representing the relationship between the number of procedures undergone by each BC patient in a timely and adequate manner and the total number of procedures recommended under the *SPSS* Medical Care Guidelines for the Management of BC, according to the clinical stage of the disease. This value was expressed within a continuous interval [0, 1]

^b^*FPGC*: Spanish acronym for Catastrophic Health Expenditure Fund

^**c**^ RPR: Relative proportion ratio

^d^ CEM: Coefficient expressing the marginal effect

## Discussion

Our results show that adherence of physicians to the Mexican Medical-Care Guidelines for Malignant Breast Tumors increased after accredited hospitals were provided with financing from the Catastrophic Health Expenditure Fund (*FPGC* by its Spanish initials) as a strategy established by law in 2007 in order to offer free treatment to women with breast cancer (BC). Analysis also indicated that this increase was higher when women were treated in early rather than in late clinical stages. After the implementation of *FPGC* accreditation, the increase in adherence was most pronounced in the prescription of expensive treatments such as trastuzumab versus other treatments. Our findings suggest that the accreditation of hospitals to receive *FPGC* financing reduced the economic barriers for BC treatment, improving medical care in certain treatment areas such as chemotherapy and trastuzumab medication, as well as in estrogen and Her2/neu receptor testing, with variance observed among hospitals.

On the downside, however, we found that a high percentage of women with positive results for estrogen and/or Her2/neu receptors remained without adequate treatment: 17.9 percent did not receive hormonotherapy and 59.4 percent were not prescribed trastuzumab medication. As reported by other studies, these findings may be attributable to a lack of knowledge among physicians regarding the *SPSS* Guidelines [7, 29]. Additionally, a shortage of supplies and/or medication (e.g. trastuzumab) could be causing a lag in treatment initiation and follow-up [29]. This issue and other *FPGC* operational problems have been highlighted by various evaluations of the *SPSS*; among other shortfalls, a delay of up to six months has been identified in the reimbursement of hospital fees by the *SPSS* to patients treated for BC [7, 29].

Our findings indicated that, notwithstanding *FPGC* financing, only 20 percent of physicians in our sample of hospitals adhered to the *SPSS* Guidelines at levels above 70 percent. Such performance appears low when compared with similar studies in various countries; in Germany, for instance, studies [14, 15, 17] have shown that between 15.7 and 67.8 percent of physicians demonstrated 100 percent adherence to the medical-care guidelines, reporting a lower percentage in cases of bilateral BC. Another study in the United Kingdom [13] reported 82 percent adherence to the BC medical-care guidelines, while a study in the Netherlands [12] reported 90.6 percent adherence for patients treated with radical mastectomy and 95 percent adherence for those managed with conservative surgery. It is important to emphasize that none of these studies allowed the recommended time between diagnosis and the beginning of treatment, an omission that could explain differences with our findings. Moreover, the above-mentioned studies were conducted in developed countries which may have benefited from more efficient systems for the provision of supplies and pharmaceuticals, a larger care package, or simply, a public health-care system different from the one in Mexico.

In contrast with our findings, Mohar et al. [3] reported that the level of adherence to BC medical-care guidelines on the part of Mexican physicians was over 80 percent. The diverging results may stem from differences in sampling: the women studied by these authors were treated in a high-specialty hospital, while our study took place in four general hospitals with very heterogeneous characteristics including differences in supplies and pharmaceutical distribution systems; the work load of health personnel; the duration of medical consultations; physician incentives; feedback on competence; knowledge about *SPSS* Guidelines; number of staff; and scale of infrastructure, among other factors [3, 21, 29]. Additionally, *FPGC*-accredited hospitals are located within geo-demographically limited areas marked by contrasting ethnic, cultural, economic, political, social, environmental and accessibility factors. The above-mentioned differences highlight the importance not only of standardizing medical-care processes, but also of improving the evaluation criteria for accrediting hospitals [7].

Our results showed that adherence to the *SPSS* Guidelines varied according to the clinical stage of the disease, with much lower performance observed in the advanced stages. This finding is consistent with those reported by other studies of women with BC [14, 15] and other types of cancer [11, 16], suggesting that variations in care at the different stages of disease could be an important factor contributing to co-morbidities, lower survival rates and increased side effects from therapeutic procedures. Our study had several strengths such as the comparison of the clinical stages and socio-demographic characteristics of patients treated before and after *FPGC* accreditation, which revealed similarities between the two groups. A second strength related to our review of the medical records of deceased patients [29], which allowed for analyzing a more representative sample. Nevertheless, the results of our study must be interpreted with caution given that analysis included women treated before and after the *FPGC*, thus possibly incorporating time-related factors–e.g., other unmeasured programs—as potential confounders. For ethical reasons, we adopted a cross-sectional, as opposed to a clinical trial, methodology for the assessment of adherence to the *SPSS* Guidelines [30].

Regarding external validity, the results of this study cannot be extrapolated to medical practice in other *FPGC*-accredited hospitals because participation was based on convenience sampling. Nonetheless, as the hospitals in our sample accounted for 48.8 percent of women with BC in Mexico, the treatment of such patients in other hospitals of this type can reasonably be expected to be similar. Our results are consistent with those reported by a nationally representative assessment of adherence conducted in Mexico in 2017 [31]. Based on interviews with 62 oncologists from *FPGC*-accredited hospitals, this study revealed a lack of knowledge about the areas of care covered or paid for by the *FPGC*, a situation clearly affecting the quality of care [31]. Also noted as major obstacles to adherence were insufficient infrastructure, equipment, personnel and supplies in terms of inputs and pharmaceuticals. These shortages were attributed to delays in the transfer of *SPSS* resources, reflecting the presence of complex and inefficient administrative processes [32].

We adjusted our analysis for the most documented confounding factor: the clinical stage of BC in our sample of patients, but not for other well documented confounders such as age [11, 12, 14, 16], schooling [21, 22], marital status [21, 33], and paid work [21], considering that these were homogenously distributed across both study groups and did not change the level of adherence when included in our regression model. Nevertheless, it is important to explore the influence of other factors associated with the patients (co-morbidities and living conditions); physicians (knowledge about *FPGC* operational processes and the *SPSS* Medical-Care Guidelines); hospitals and/or health programs (delay in medical care and/or therapeutic procedures, and availability of supplies and pharmaceuticals); and infrastructure and equipment (adequacy and conditions). These should be taken into account in future efforts to determine the impact of *FPGC* accreditation on the adherence of physicians to the *SPSS* Medical-Care Guidelines for Malignant Breast Tumors.

## Conclusion

Results from this study suggest that subsidizing the cost of treatment for BC and other diseases at accredited hospitals boosts the adherence of physicians to medical-care guidelines. However, it is essential to streamline the processes for the transference of funds and to implement other innovative strategies in pursuit of greater levels of adherence on the part of physicians. This will contribute to the delivery of timely and efficient medical care according to the guidelines established by the Mexican Health-Care System and result in a higher quality of life and lower mortality rates among women with BC.

## Supporting information

S1 Table Level of adherence^a^ to the Medical-Care Guidelines for Malignant Breast Tumors, by characteristics of women treated before and after *FPGC*^b^ accreditation of the four participating hospitals, Mexico. ^a^The index of adherence was evaluated in a bivariate manner, estimating means and 95 percent confidence intervals (CI) in women treated before and after *FPGC* accreditation. Adherence level was a numerical value contained in the continuous interval [0, 1], which expressed the relationship between the number of procedures undergone by the BC patient in a timely and correct manner and the total number of procedures specified in the *SPSS* Medical-Care Guidelines for Malignant Breast Tumors, according to the clinical stage of disease in each patient.
^b^*FPGC*: Spanish acronym for Catastrophic Health Expenditure Fund.
^c^Means and confidence intervals of adherence levels were expressed as proportions in relation to the study variables and whether the patient was treated before or after *FPGC* accreditation.
(PDF)
