# Supplementary material for: Adherence of Mexican physicians to clinical guidelines in the management of breast cancer: Effect of the National Catastrophic Health Expenditure Fund
Source: PLoS One. 2019 Mar 20;14(3):e0212841. doi: 10.1371/journal.pone.0212841 (PMC6426232; doi:10.1371/journal.pone.0212841)
Supplement: S1 Table — aThe index of adherence was evaluated in a bivariate manner, estimating means and 95 percent confidence intervals (CI) in women treated before and after FPGC accreditation. Adherence level was a numerical value contained in the continuous interval [0, 1], which expressed the relationship between the number of procedures undergone by the BC patient in a timely and correct manner and the total number of procedures specified in the SPSS Medical-Care Guidelines for Malignant Breast Tumors, according to the clinical stage of disease in each patient. bFPGC: Spanish acronym for Catastrophic Health Expenditure Fund. cMeans and confidence intervals of adherence levels were expressed as proportions in relation to the study variables and whether the patient was treated before or after FPGC accreditation. (PDF) [file pone.0212841.s001.pdf]

Supporting information

S1 Table. Level of adherence[a] to the Medical-Care Guidelines for Malignant Breast Tumors, by characteristics of women treated before and after the accreditation of four participating to receive *FPGC*[b] financing, Mexico.

	Before *FPGC*[b] n=212	p value	After *FPGC*[b] n=267	p value	Total n=479	p value
	Means [95% Confidence interval][c]					
**General**	0.420 [0.387 - 0.453]	<0.001	0.496 [0.466 - 0.525]	0.001	0.462 [0.440 - 0.485]	<0.001
**>70% adherence**	0.184 [0.131 - 0.237]	<0.001	0.202 [0.155 - 0.250]	0.616	0.194 [0.159 - 0.230]	<0.001
**Characteristics of women**						
**Clinical stage of breast cancer**						
0	0.667 [0.305 - 1.000]	<0.001	0.611 [0.442 - 0.780]	<0.001	0.625 [0.461 - 0.789]	<0.001
I and IIA	0.489 [0.422 - 0.556]	0.342	0.609 [0.567 - 0.651]	0.979	0.563 [0.526 - 0.601]	0.473
IIB and III	0.408 [0.365 - 0.451]	0.163	0.426 [0.392 - 0.459]	0.035	0.417 [0.390 - 0.444]	0.014
IV	0.181 [0.033 - 0.328]	0.015	0.417 [0.306 - 0.527]	0.059	0.308 [0.217 - 0.399]	0.001
**Age groups**						
<40	0.384 [0.251 - 0.517]	<0.001	0.473 [0.378 - 0.568]	<0.001	0.436 [0.356 - 0.515]	<0.001
40-49	0.437 [0.369 - 0.506]	0.487	0.506 [0.457 - 0.555]	0.544	0.477 [0.436 - 0.518]	0.364
50-59	0.419 [0.354 - 0.485]	0.641	0.453 [0.404 - 0.502]	0.720	0.438 [0.398 - 0.478]	0.953
60-69	0.411 [0.316 - 0.507]	0.746	0.556 [0.493 - 0.619]	0.151	0.501 [0.446 - 0.555]	0.183
≥70	0.416 [0.331 - 0.501]	0.691	0.502 [0.422 - 0.582]	0.641	0.454 [0.396 - 0.513]	0.708
**Schooling**						
None	0.399 [0.333 - 0.466]	<0.001	0.510 [0.457 - 0.564]	<0.001	0.457 [0.415 - 0.499]	<0.001
Elementary	0.411 [0.345 - 0.477]	0.810	0.470 [0.416 - 0.525]	0.305	0.441 [0.399 - 0.484]	0.609
Junior high school	0.442 [0.348 - 0.536]	0.470	0.496 [0.440 - 0.552]	0.712	0.478 [0.428 - 0.528]	0.533
High school	0.452 [0.356 - 0.547]	0.378	0.541 [0.474 - 0.608]	0.481	0.505 [0.449 - 0.561]	0.184
Professional	0.431 [0.315 - 0.547]	0.643	0.436 [0.340 - 0.531]	0.180	0.433 [0.359 - 0.508]	0.587

Continued

S1 Table…*Continued*

	Before *FPGC*[b] n=212	p value	After *FPGC*[b] n=267	p value	Total n=479	p value
			Means [Confidence interval 95%][c]			
**Marital status**						
Consensual union	0.564 [0.437 - 0.691]	<0.001	0.453 [0.341 - 0.565]	<0.001	0.510 [0.425 - 0.595]	<0.001
Separated/divorced/widow	0.402 [0.322 - 0.482]	0.035	0.513 [0.454 - 0.572]	0.351	0.466 [0.417 - 0.515]	0.377
Married	0.406 [0.357 - 0.454]	0.023	0.507 [0.467 - 0.546]	0.375	0.459 [0.428 - 0.490]	0.267
Single	0.420 [0.331 - 0.509]	0.069	0.469 [0.412 - 0.525]	0.809	0.451 [0.402 - 0.501]	0.240
**Paid work**						
No	0.406 [0.358 - 0.454]	<0.001	0.507 [0.471 - 0.544]	<0.001	0.462 [0.433 - 0.492]	<0.001
Yes	0.439 [0.384 - 0.495]	0.366	0.480 [0.439 - 0.522]	0.338	0.462 [0.428 - 0.497]	0.999
			**Treatment characteristics**			
**Type of treatment**						
Conservative surgery	0.482 [0.397 - 0.568]	0.113	0.516 [0.440 - 0.591]	0.581	0.498 [0.441 - 0.556]	0.178
Radical surgery	0.480 [0.436 - 0.523]	<0.001	0.527 [0.495 - 0.559]	<0.001	0.507 [0.481 - 0.534]	<0.001
Chemotherapy	0.468 [0.429 - 0.508]	<0.001	0.494 [0.464 - 0.523]	0.688	0.483 [0.459 - 0.507]	<0.001
Radiotherapy	0.475 [0.430 - 0.519]	<0.001	0.485 [0.451 - 0.520]	0.332	0.481 [0.452 - 0.509]	0.039
Hormonotherapy	0.479 [0.430 - 0.528]	0.001	0.538 [0.502 - 0.574]	0.000	0.513 [0.484 - 0.542]	<0.001
Trastuzumab	0.480 [0.244 - 0.716]	0.613	0.548 [0.480 - 0.616]	0.103	0.541 [0.470 - 0.611]	0.021
			**Medical care characteristics**			
**Hospital**						
South	0.056 [0.000 - 0.265]	0.602	0.313 [0.190 - 0.435]	<0.001	0.227 [0.116 - 0.338]	<0.001
North	0.391 [0.333 - 0.449]	0.003	0.450 [0.410 - 0.489]	0.037	0.426 [0.392 - 0.460]	0.001
West	0.455 [0.354 - 0.556]	0.001	0.618 [0.561 - 0.675]	<0.001	0.566 [0.514 - 0.619]	<0.001
Center	0.455 [0.404 - 0.506]	<0.001	0.504 [0.457 - 0.550]	0.004	0.477 [0.442 - 0.512]	<0.001

[a]The index of adherence was evaluated in a bivariate manner, estimating means and 95 percent confidence intervals (CI) in women treated before and after *FPGC* accreditation. Adherence level was a numerical value contained in the continuous interval [0, 1], which expressed the relationship between the number of procedures undergone by the BC patient in a timely and correct manner and the total number of procedures specified in the *SPSS* Medical-Care Guidelines for Malignant Breast Tumors, according to the clinical stage of disease in each patient.
[b]*FPGC*: Catastrophic Health Expenditure Fund.
[c]Means and confidence intervals of the adherence level expressed as proportions in relation to the study variables and whether the patient was treated before or after *FPGC* accreditation.
